# A Therapeutic Success Story of Lucio’s Phenomenon: 10 Years After

**DOI:** 10.4269/ajtmh.26-0055

**Published:** 2026-07-07

**Authors:** Gerardo Alvarado-Villa, Jesús Salvador Velarde-Félix

**Affiliations:** ^1^ Clínica de Consulta Familiar del ISSSTE y Centro de Salud Urbano Guasave (IMSS Bienestar), Guasave, Mexico;; ^2^ Hospital General de Culiacán, Servicios de Salud de Sinaloa (IMSS Bienestar), Culiacán, Mexico;; ^3^ Lic. en Biomedicina, Universidad Autónoma de Sinaloa, Culiacán, Mexico

**Figure 1.**
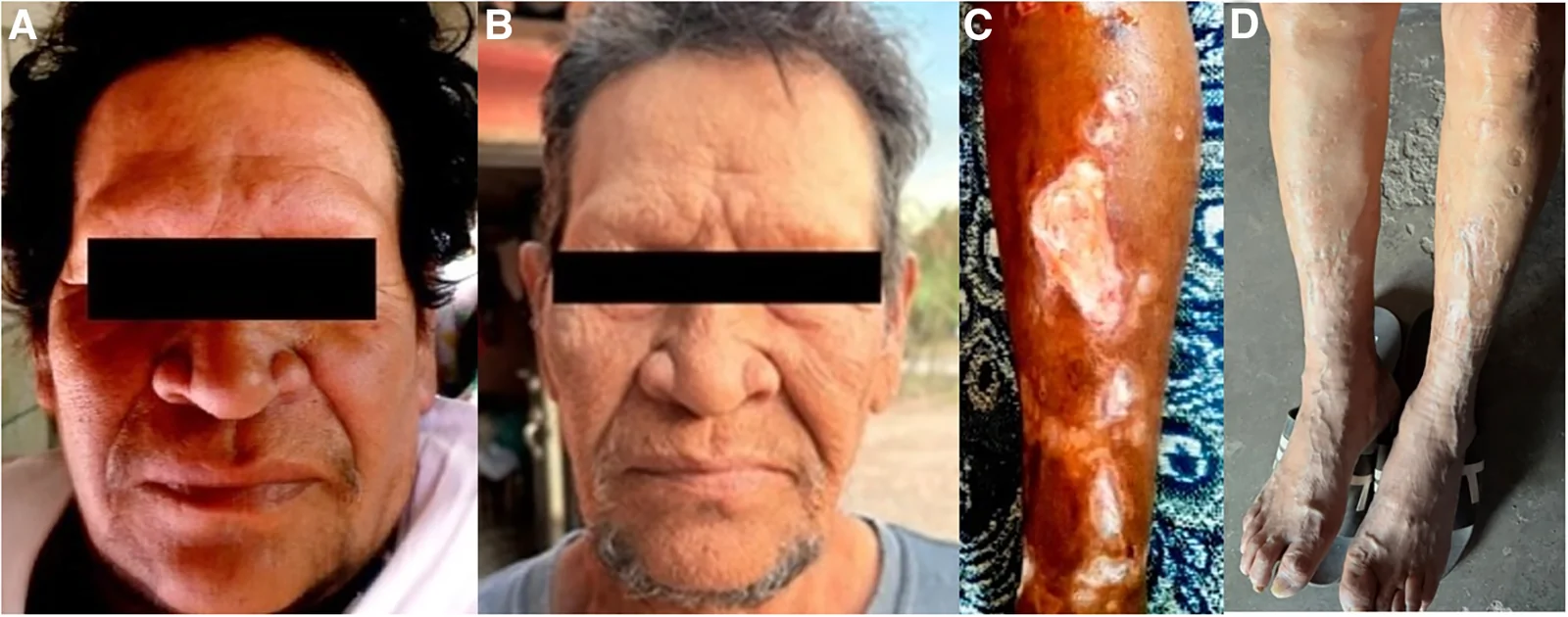
Comparison of lesions before and after treatment. (**A**) Diffuse infiltrative leonine facies with total madarosis (previously published image).^1^ Now, after 10 years, the diffuse infiltrative and leonine facies have disappeared. (**B**) Skin discoloration associated with clofazimine resolved, but the complete madarosis remains visible. (**C**) Left leg with multiple injuries (previously published image).^1^ (**D**) Skin ulcers in both lower extremities and erythema nodosum leprosum are healed. Instead, only traces (scars) of the injuries can be observed on both legs.

A 60-year-old man born in Sinaloa, Mexico, was diagnosed with Lucio’s phenomenon (LP) associated with *Mycobacterium lepromatosis* (*M. lepromatosis*) infection (Figure 1), previously published in this journal in 2016 by our research team.^1^

Lucio’s phenomenon is an immunological reaction observed in patients with the diffuse form of lepromatous leprosy. It manifests as diffuse skin infiltration, erythematous purpuric macules, irregular skin ulcers, skin necrosis, lymphohistiocytic inflammatory infiltrate, thrombosis, vascular occlusion, and invasion of blood vessels by leprosy bacilli.^2^

Fortunately, the patient responded appropriately to a 2-year course of World Health Organization-recommended multidrug treatment of multibacillary leprosy with clofazimine, rifampicin, and dapsone, beginning in May 2015.^1^

Additionally, he received adequate medical home care consisting of daily cleaning of severe skin ulcers with soap and water, followed by application of a homemade natural ointment for wounds (a non-commercial dermatological preparation with moisturizing, antiseptic, and anti-inflammatory properties that contains petrolatum, lanolin, zinc oxide, and talc). As treatment of erythema nodosum leprosum (ENL), thalidomide therapy was administered for 20 days at a dose of 200 mg daily, which was well tolerated. The fever and pain, typical of ENL, subsided 2 days post-treatment. To prevent secondary bacterial infections, dicloxacillin was prescribed at a dosage of 500 mg in two prophylaxis cycles, each lasting 20 days separated by 10 days, and diclofenac was administered at a dosage of 50 mg for 10 days to reduce pain and fever. The pharmacological therapy was corticosteroid-free.

After the diagnosis was established, bacilloscopy of the ear and histopathology were performed twice more (every 6 months after the initial diagnosis), confirming the mycobacterial infection. For this reason, the treatment was extended until July 2017. Finally, a histopathological study conducted in December 2017 yielded a negative result for *M. lepromatosis*. Additionally, the peripheral neuropathy in the patient (thickening of the ulnar and peroneal nerves, paresthesia in the plantar region, and sensory loss in both extremities) resolved. Now, the patient remains free of injuries and disabilities and works as a herder.

Given the low occurrence and lack of predictive or prognostic measures for LP, high morbidity, such as physical disabilities or amputation, may occur; moreover, standardized therapeutic measures for this clinical variant of leprosy reaction do not exist. In line with other authors who have achieved therapeutic success without administering corticosteroids,^3^^,^^4^ only standard multibacillary multidrug therapy in combination with home care–a non-commercial dermatological preparation and diclofenac, both with anti-inflammatory properties–were used. Mexico is one of the countries with the highest records of LP,^5^ specifically in Northern Mexico, where Sinaloa state is localized, which remains historically the Mexican region with the highest number of registered patients with leprosy.^6^
